# 

**Published:** 1897-01

**Authors:** 


### CONTENTS.

ORIGINAL COMMUNICATIONS. PAGE

Hypersensitivity of Dentine and its Treatment. By HENRY H. BURCHARD, M.D., D.D.S., Philadelphia . . . . 1

The Value of Statistics in Cataphoresis. By L. E. CUSTER, B.S., D.D.S., Dayton, Ohio . . . . 11

A Study of the Relation of the Frontal Sinus to the Antrum. By DR. THOMAS FILLEBROWN, Boston, Mass. . . . . 14

ABSTRACTS AND TRANSLATIONS.

A Method of Strengthening Vulcanite Plates. By C. R. MORLEY, L.D.S. (Eng.) 19

REPORTS OF SOCIETY MEETINGS.

American Dental Association . . . . 21

Academy of Stomatology . . . . 30

The New York Institute of Stomatology . . . . 40

Central Dental Association of Northern New Jersey . . . . 43

EDITORIAL.

The Needs of the Future . . . . 58

Correction of Dr. Richards's Paper . . . . 61

BIBLIOGRAPHY . . . . 62

DOMESTIC CORRESPONDENCE.

Calculus in Sublingual Gland . . . . 63

Experiments in Cataphoresis . . . . 64

CURRENT NEWS.

The International Tooth-Crown Company *versus* Allan G. Bennett . . . . 65

Pennsylvania Association of Dental Surgeons . . . . 68

The New York Institute of Stomatology . . . . 68

